# Review of Stratum Corneum Impedance Measurement in Non-Invasive Penetration Application

**DOI:** 10.3390/bios8020031

**Published:** 2018-03-26

**Authors:** Fei Lu, Chenshuo Wang, Rongjian Zhao, Lidong Du, Zhen Fang, Xiuhua Guo, Zhan Zhao

**Affiliations:** 1 State Key Laboratory of Transducer Technology, Institute of Electronics, Chinese Academy of Sciences, Beijing 100190, China; lufei_flyer@163.com (F.L.); wangchenshuo16@mails.ucas.ac.cn (C.W.); zhaorongjian15@mails.ucas.ac.cn (R.Z.); lddu@mail.ie.ac.cn (L.D.); zfang@mail.ie.ac.cn (Z.F.); 2 University of Chinese Academy of Sciences, Beijing 100049, China; 3 School of Public Health, Capital Medical University, Beijing 100069, China; guoxiuh@ccmu.edu.cn; 4 Beijing Municipal Key Laboratory of Clinical Epidemiology, Beijing 100069, China

**Keywords:** mobile medical care, Non-invasive monitoring, skin impedance, skin permeability, Impedance model

## Abstract

Due to advances in telemedicine, mobile medical care, wearable health monitoring, and electronic skin, great efforts have been directed to non-invasive monitoring and treatment of disease. These processes generally involve disease detection from interstitial fluid (ISF) instead of blood, and transdermal drug delivery. However, the quantitative extraction of ISF and the level of drug absorption are greatly affected by the individual’s skin permeability, which is closely related to the properties of the stratum corneum (SC). Therefore, measurement of SC impedance has been proposed as an appropriate way for assessing individual skin differences. In order to figure out the current status and research direction of human SC impedance detection, investigations regarding skin impedance measurement have been reviewed in this paper. Future directions are concluded after a review of impedance models, electrodes, measurement methods and systems, and their applications in treatment. It is believed that a well-matched skin impedance model and measurement method will be established for clinical and point-of care applications in the near future.

## 1. Introduction

Compared with many other complex technologies in the biomedical engineering field, bioimpedance detection is a fast, simple, non-invasive, and cost-effective method for assessing the condition of the human body [1]. So far, it has been applied to many fields, such as heart rate measurement [2], fake finger detection in security systems [3], dermatological research [4], and so on. However, only a few applications have been realized after six decades of development. This is due to the difficulty of collecting an accurate signal and finding a one-to-one match between impedance and physiological information, due to the lack of appropriate detection electrodes as well as the complexity of, and individual differences between, bodies. 

Skin impedance is an important facet of bio-impedance and can be used to analyze the condition of the human body. Its measurement can feed back physiological information about the skin, such as skin hydration [5], the thickness of the stratum corneum [6,7], the condition of water channels through the skin [8,9], and so on. In addition, electrode–skin impedance is a significant factor in the application of other bioimpedance parameters, such as heart rate measurement, which greatly affects the target signal quality [10]. Detailed research of skin impedance has been conducted on a combination of skin characteristics and bioimpedance parameters [11] and dates back to the 1950s, mainly driven by transdermal drug delivery technology. Basically, skin impedance is the response of a specific skin region to an externally applied electrical current (or voltage) [11]. Therefore, the electrode is crucial for the detection of skin impedance. The measurement of skin impedance is increasingly required in the research of non-invasive disease monitoring, such as diabetes and gout [12]. The GlucoWatch G2TM Biographer (“GW2B” Cygnus, Inc., Redwood, CA, USA) was approved for monitoring blood glucose non-invasively by transdermal ISF extraction in 2002 [13]. However, two problems—skin irritation and uncertainty of blood glucose measurement—have arisen from its wearers, which caused its failure as a product. Partly, the reason for these problems was that the wearable device could not distinguish between different individuals’ skin conditions, which can be assessed by skin impedance analysis. Heretofore, skin impedance measurement has not been fully developed, especially for the stratum corneum (SC).

Skin permeability characterization, especially in the assessment of the penetration promotion effect, is one of the most important applications of skin impedance. The relationship between skin permeability and skin impedance has been studied by various methods [11,14,15,16,17], and much effort has been put towards proving the relationship between skin penetration and skin impedance, especially for the impedance of SC. References [18,19] demonstrated that humidity has a great influence on skin impedance because it changes the hydration state of SC. Some researchers have studied the barrier function of SC by serial tape stripping and found that the thicknesses of SC among different individuals are varied [6]. Meanwhile, detected skin impedance is closely related to the thickness of SC. Also, the impedance of SC occupies a huge proportion of the total skin impedance. Thus, the status of SC greatly affects the detected skin impedance. Reference [20] evaluated the effect of penetration enhancers on transdermal drug delivery by comparing skin impedance spectroscopies. Penetration enhancers are used to open penetration channels through skin, especially for breaking the barrier function of SC and promoting skin hydration; thus, they can reduce skin impedance. Therefore, skin impedance detection is a simple, fast and non-invasive method to feed back the effect of penetration enhancers. In addition, electroporation, another commonly used penetration method, was used as pretreatment for transdermal drug delivery in reference [21]. It was pointed out that skin impedance detection could be used to reflect the effect of electroporation. In reference [11], researchers used a measurement technique based on Electrical Impedance Spectroscopy (EIS) aimed at discriminating the electrical characteristics of the electrical treatment for human skin before drug delivery. An electrical model of skin was proposed to allow researchers to analyze physiological changes from the perspective of electrical technology. Hence, the changes in each parameter in the model, which represent different physiological meanings, have also been studied. However, researchers have not fully explored the mathematical equation between skin permeability and impedance. However, it is feasible to use the SC impedance measurement instead of skin permeability due to its convenience and cost-effectiveness. Therefore, the development of a skin impedance measurement will be reviewed in this paper to analyze the assessment of skin permeability.

Many researchers have tried to use skin impedance to assess skin penetration ability [18]. It has been pointed out that the electrode, analysis model and measurement method are the key factors in this process. Thus, the theoretical model, choice of electrodes and method of skin impedance measurement will be reviewed to forecast the future development of skin impedance measurement. This will allow improved accuracy of quantitative ISF extraction in non-invasive disease monitoring techniques as well as the amount of drug absorption in transdermal drug delivery. The content includes the following four parts:

1. Skin impedance model;
2. Electrodes for skin impedance measurement;
3. Methods and system for impedance measurement;
4. Application in penetration promotion treatment.

## 2. Skin Impedance Model

The ideal sites for impedance measurement in a in vivo experiment are the human forearm and side abdomen, because they can be conveniently accessed and have a relatively thin SC [6]. The mammalian abdomen is preferred for in vitro experiments due to its thin property and large area [22]. In order to clearly disclose the model of skin impedance, it is necessary to figure out the anatomy of skin. Here, firstly, the skin structure will be introduced from the outermost to the innermost skin layer. Then, the transport physical principles of skin are discussed. Finally, the skin impedance model is studied.

### 2.1. Morphology of Human Skin

**Figure 1:**
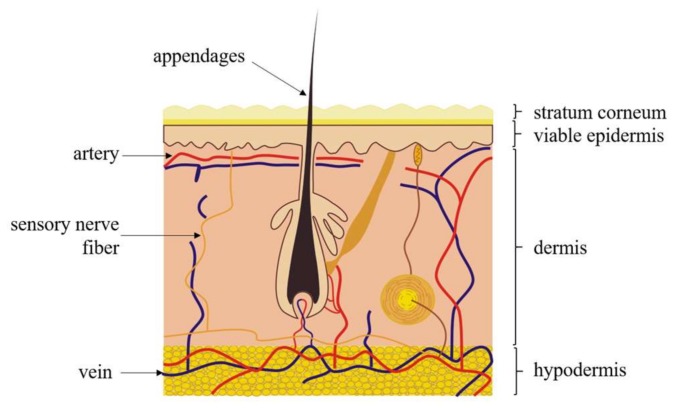
Sketch of the human skin layers. Moving from the outside to the inside: the stratum corneum (the outmost layer), the viable epidermis and the dermis [23].

Mammalian skin plays a great role in protecting the body [23]. The general human skin consists of three major layers, including SC, the viable epidermis and the dermis (Figure 1). Also, there are some skin appendages in the horizontal direction, like sweat glands and hair follicles [24].

The outermost layer is the stratum corneum, which is considered to be the biggest barrier in transdermal transport [25]. The thickness of SC varies greatly among different individuals and different body parts. It is recorded that the thickness of the forearm is about 10~40 μm [7,26]. SC is mainly composed of corneocytes which are dead cells without nuclei [25] that are embedded into the highly ordered and dense lipid matrix. The moisture content of SC is kept at about 20 percent. It is believed that such a structure and composition determines the hydrophobic properties of skin [22] as well as the narrow transport channel, which turns out to have high skin impedance [23]. Basically, high skin impedance caused by the SC layer can be measured when the frequency is below 1000 Hz [27]. Furthermore, saturated lipids can be another permeability barrier, such as ceramides, cholesterol and free fatty acids [28]. Defects of SC may affect the transdermal transport, but the extent to which this occurs is not very clear yet. In conclusion, the SC properties will greatly affect the measurement of skin impedance and the evaluation of skin permeability due to its special physiological structure, which deserves to be studied. 

The layer below SC is called the viable epidermis, which is compared with that of the differentiated dead cells in SC [24]. The thickness of the viable epidermis also varies greatly, and the average thickness (except for special parts like palm and sole) is about 150 μm [23]. The viable epidermis is composed of epithelial cells, follicular epidermis and some related appendages, like hair follicles, sebaceous glands and sweat glands [29]. It can be divided into four layers vertically and they are the stratum lucidum, stratum granulosum, stratum spinosum, and stratum basale [23]. There are no blood vessels in the viable epidermis, where protein occupies 40 percent, water occupies around 40 to 60 percent and lipid occupies 15 to 20 percent. Hence, the viable epidermis is a hydrophilic layer [30].

Below the viable epidermis is the dermis layer, which may have a thickness about 500–2000 μm, depending on the body site [23]. The outmost part is the papillary dermis, consisting of thin collagen bundles, elastic fibers, fibrocytes and ground substance, comprising mainly water, electrolytes, polysaccharides–polypeptide complexes, and plasma proteins. Most of the microvascular system is located in this layer. The blood vessel area increases with depth from the epidermal–dermal junction. The dermis houses blood vessels, lymphatics and some skin appendages, as well as the nervous system within the skin. A large number of ISF are distributed in the dermis layer. In transdermal drug delivery applications, drugs are supposed to be preliminarily transported into the dermis and then into blood vessels. Subcutaneous fat tissue, which lies underneath the dermis, is about several millimeters thick. The water content of the dermis is about 60%, which is similar to that of the epidermis [30]. The dermis and the viable epidermis, which are together called the viable skin layer, are similar in electrical properties and water composition. The characteristics of viable skin are mainly observed at a high frequency, especially above 1 MHz [27]. To conclude, the properties of each skin layer are summarized in Table 1.

The hair follicle has a conical entrance and extends approximately 500 μm deep from the skin surface to the sebaceous duct that connects the follicle with the sebaceous gland. The gland produces sebum, a lipophilic substance which is composed of triglycerides, wax, squalene, cholesterol and lipids, and fills the funnel shaped tube. The lipophilic substance protects skin against bacteria, excessive moisture and heat loss. The sweat glands secrete water and evaporate to give a lower body temperature and moisturize skin. Its root is located in the lower dermis or subcutaneous tissue and is connected with the skin surface by a duct 100 μm in diameter, rising straight through the dermis and spiraling up the epidermis [24]. Electrolytes and water can be absorbed through the duct wall to maintain homeostasis. There are also some other endocrine glands and eccrine glands that work similarly to sweat glands.

### 2.2. Physical Principles of Skin Transport

**Figure 2:**
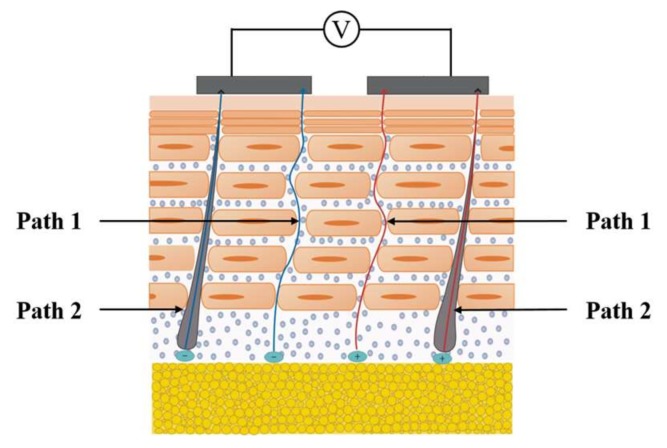
Schematic of the two transport paths for ions in dermis. Path 1 represents ions moving along the dermis–viable epidermis–SC through the intercellular lipid bilayer path; path 2 represents ion transport directly through the sweat glands to the skin surface.

The study of the transport mechanisms of charged ions in the skin could help to combine physiological changes with electrical performance. There are two main paths for charged ions to be transported to the skin surface: dermis–viable epidermis–SC, and skin appendages (here, thisrefers to sweat glands) (Figure 2).

As for the dermis–viable epidermis–SC path, the transport of ions in the dermis is similar to that in aqueous media [24]. The main resistance comes from the aqueous medium, while the contraction and expansion of the blood vessels could provide a certain driving force as well as an ion source for the transport process. The ions’ transport pattern in the viable epidermis is similar to that in the dermis, where the resistance is determined mainly by the aqueous medium and partly due to the proteins and crossed cell membranes [31]. In the stratum corneum, there are two routes for ions to move, one of which is the intercellular lipid bilayer path and the other is the carrier protein in transcellular transport [32]. However, according to the microscopic results and SC transport model, the intercellular lipid bilayer path is the main route [33]. Ions move least easily in the SC due to the hydrophobic nature and dense structure of this layer. Thus, the impedance of SC is much larger than the other two layers [34]. Therefore, SC presents the greatest barrier for ion transdermal transport.

In the sweat glands, the ions in the dermis or subcutaneous tissue diffuse into the roots of the sweat glands and are transported through the duct to the skin surface. They can avoid the huge resistance caused by the stratum corneum, but the degree of ion transport through this way is not clear yet [16].

### 2.3. Skin Impedance Model

Basically, there are two types of electrical properties found in the analysis of skin impedance data, and they are resistance (R) and capacitance (C). Thus, the skin impedance is composed of a real part and an imaginary part, which represent the skin resistance and capacitance properties, respectively. The value of each part is mainly related to the structure and composition of skin [29]. Hair follicles and sweat glands exhibit the resistance property, while the lipid bilayer shows the capacitance property [24]. A skin impedance model was constructed to provide a standard electrical method for researchers to study regarding the physiological changes of the skin, allowing the microstructure and composition changes of skin to be characterized quantitatively by model parameters. According to the literature, current skin impedance models can be divided into two categories: constant phase angle model and RC layered model. The former focuses on the biological characteristics of the skin while the latter considers the nature of physiological stratification.

#### 2.3.1. Constant Phase Angle Model

**Figure 3:**
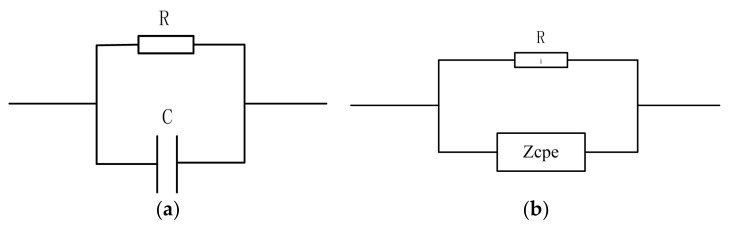
Two simple impedance models: (**a**) represents the initial RC skin impedance model; (**b**) represents the improved model based on (**a**) and considers the biological characteristics of the skin; the capacitance element was replaced by the constant phase angle element.

The initial skin impedance model is a simple R-C circuit in parallel or series, as shown in Figure 3a. However, a single RC model cannot express the complex structure of skin. References [11,20] proposed an improved model, the constant phase angle (CPA) model, which adopts the constant phase angle element, Z_cpe_.

The CPA model is based on the well-known biological impedance model used by Cole in 1940 [35]. Z_cpe_ was added into the conventional RC circuit in parallel or in series; the simplest version involves the replacement of capacitance with Z_cpe_, as shown in Figure 3b. Z_cpe_ is an empirical function commonly used in fitting circuits for impedance spectroscopy measurement, and its mathematical expression is shown in Equation (1):

(1) $$Z_{\mathrm{cpe}}{=A(j\omega )}^{-\alpha }$$

where, Z_cpe_ represents a pseudo capacitor, A is a constant that represents the magnitude of the quasi capacitance impedance. Z_cpe_ is a pure capacitance when α = 1, while it is a pure resistance when α = 0. α is a parameter which is closely related to skin properties like water content. For common human skin, α is considered to be 0.8 [36]. The Warburg element, Z_w_, is a special constant phase angle model when α = 0.5, whose mathematical expression is shown in Formula (2). It has been commonly used in impedance models of sweaty skin or skin with a large water content instead of Z_cpe_ to simplify the model fitting calculation [37].

(2) $$Z_{w}{=A(j\omega )}^{-0.5}$$

The skin, as a part of living organisms, consists of lots of living cells. The CPA model can reduce the error caused by the dispersion effect of cell membranes, as well as the nonuniformity of electrode surfaces and the inhomogeneity of skin [38,39]. The data fitted by the constant phase angle model was proven to be closer to the true curve of the measured impedance spectrum than the initial single RC model [20]. However, the superiority was found to be very limited, which did not work very effectively in cases where penetration enhancers were used.

The CPA model is based on the traditional RC model, taking into account the biological characteristics of the skin while ignoring its layered nature. The biological and chemical properties of each layer are not the same—the skin is anisotropic as well. Thus, the constant phase angle model is not accurate enough to express the electrical properties of skin.

#### 2.3.2. RC Layered Model

In recent years, with researchers exploring the microscopic structure of skin deeply, they have considered the hierarchical structure of skin and have constructed a layered skin impedance model.

**Figure 4:**
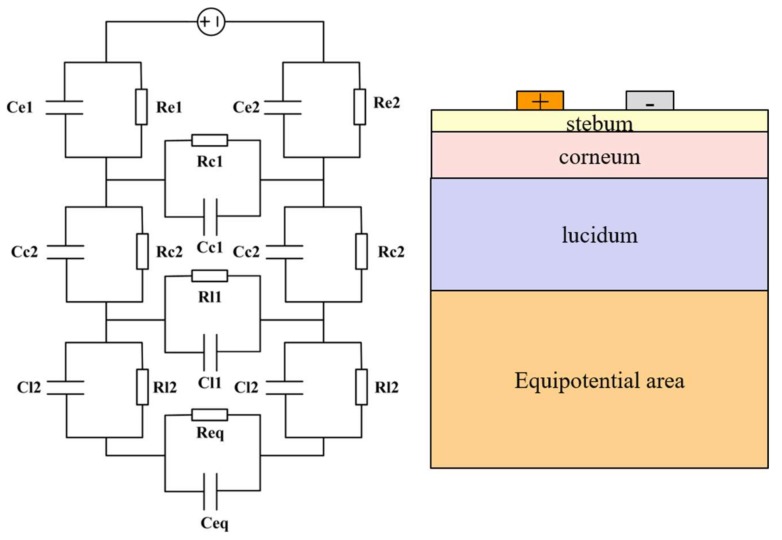
Equivalent circuit of human skin impedance measurement, which considers the layered nature of the skin, so the layered impedance was built vertically as well as the interlayer skin impedance in the horizontal direction [40].

The authors of study [40] proposed a new skin impedance model (Figure 4), which uses a thin film non-symmetric micro-electrode array for skin impedance measurement. The model constructed in that paper not only considered the layered impedances vertically, but also took into account the interlayer impedances in the horizontal direction. The interlayer impedances are the responses of the part of current only flowing through each layer vertically. In addition, the contact impedance between electrode and skin was considered in this model.

**Figure 5:**
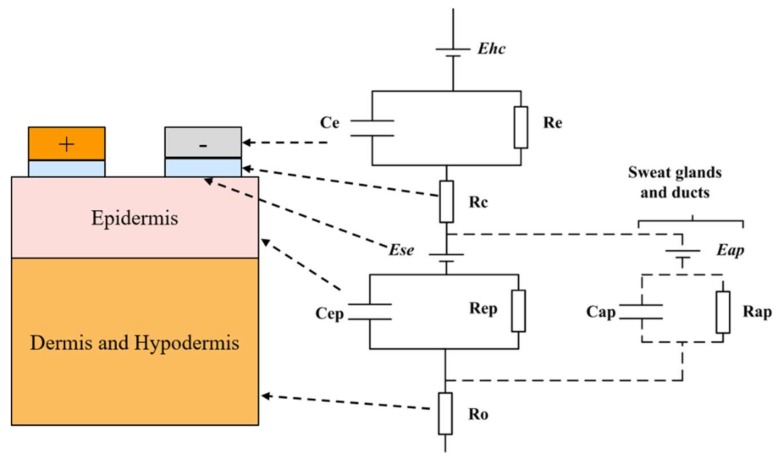
Equivalent circuit of human skin impedance measurement, which stressed the layered structure and the skin appendages [12].

This modeling concept was proposed again in reference [12] (Figure 5), based on the longitudinal layered structure of the skin. Apart from the impedance effects of each layer, these authors also considered the impedance caused by skin appendages, such as sweat glands and ducts.

The skin could be divided into three layers vertically—if we consider each layer as uniform, the skin can be modeled as three layers of homogeneous materials of different properties stacked closely. This layered modeling method would make the overall fitting results show better accuracy. The different measured impedance data can be reflected into the specific changes in each specific layer, and thus it is easier for us to deduce the refined physiological structure changes of skin. However, the number of elements and unknown parameters in the RC layered model is much larger than those of the traditional RC model and CPA model, bringing a large amount of calculation in later data analysis. This is why layered models have been only in concept form in the literature. In addition, the basic units of the layered model are RC units in parallel, which cannot characterize the biological characteristics of the skin, such as the dispersion effect of cell membranes. In fact, the skin layer is not uniform, nor is the electrode surface; thus, the non-uniformity will lead to non-uniform current density. 

To conclude, the characteristics of the constant phase angle model and the RC layered model are listed and compared in Table 2. Combining the characteristics of the two models analyzed above, a new type of model should be constructed to characterize both the biological properties and layered structure of skin. The RC layered structure should adopt the Z_cpe_ element in each layer module to compensate for the differences in diffusion. Two methods could be provided to reduce the complexity and calculation of the model. Firstly, the two layers of viable epidermis and dermis could be merged into viable skin layer because the electrical properties of the two layer are similar compared with the high resistive SC. Secondly, Z_w_ could be used instead of Z_cpe_ because the skin parts studied for ISF extraction or transdermal drug delivery, like the forearm, upper arm and abdomen [22,41], contain relatively large water contents. In addition, with the advances in computing technology, the more complex problems of model parameter fitting should be solved in the near future.

## 3. Electrodes for Skin Impedance Measurement 

As a kind of the electrophysiological signal, skin impedance has the characteristics of a weak, large interference and a low signal to noise ratio. Normally, the impedance signals collected by electrodes include the skin–electrode contact impedance as well as the impedance of the skin itself. Thus, it is most important to choose suitable electrodes as the impedance detection sensors. This section introduces three aspects of electrode selection criterion, including types, materials and geometry.

### 3.1. Types of Electrodes

Electrodes that are used for biological signal detection can be broadly classified into two categories—wet electrode, dry electrode and others [42,43]—as well as the skin impedance measurement. Both of the two types of electrodes serve as transducers to convert the ionic current in the detected body to the electrical current in the electrode.

A wet electrode is more like a nonpolarizable electrode and can be divided into two types as well: pre-gelled electrode and electrolyte electrode [17,44]. The pre-gelled electrode, which is generally referred to as the disposable pre-gelled Ag/AgCl wet electrode, is more convenient to use and is preferred in electrophysiological signal detection due to its low cost, good fitting with skin and nice conductivity. However, the wet electrode has two significant disadvantages: it changes the electrical properties of skin by ions from gel or electrolyte transporting into skin as well as SC overhydration. In addition, the contact between electrodes and skin would become worse due to the accumulation of sweat and grease over long time use; the gel conductivity would also worsen. Therefore, the wet electrode is only suitable for single use, short time signal measurement.

A dry electrode is more like a polarizable electrode due to its large contact capacitance. It is generally referred to as electrodes that are attached to the skin surface directly without a gel layer or electrolyte, and the material is mainly based on metal [42]. Due to the lack of gel layer, the electrode usually cannot be adhered to the skin surface completely, especially for rigid electrodes. Thus, air exists between the electrode and the skin surface in the form of a dielectric layer, which greatly increases the total impedance. The electrode–skin interface impedance largely depends on the pressure and humidity. The pressure applied to the electrode could improve the contact and thus the impedance would be reduced. The improved humidity could improve the hydration and conductivity of the skin [45]. In addition, the humidity would change because of the accumulation of sweat, which would cause changes in the conductivity of the dielectric layer [18]. In order to reduce the impact on the skin impedance measurement, we should allow sufficient steady time before detection. Dry electrodes are generally made from hard material and are difficult to attach well to the skin surface. This leads to high contact resistance and motion artifacts, in addition to an unfixed skin–surface contact area. One generally adopted solution for this problem is to take some measures to soften the electrode, such as use soft substrates like flexible polyimide, Polydimethylsiloxane (PDMS) and textile [46,47,48].

In addition, several new forms of electrodes have been developed in recent years, such as tattoo-based electrodes [49] and skin-like electrodes [50], which are supposed to be appropriate for biological signal detection. These soft electrodes could provide better attachment with human skin and reduce the discomfort for wearers. Therefore, in the application of non-invasive disease monitoring by ISF and transdermal drug delivery, it is predicted that the sensors would be preferred in the soft form for SC impedance detection.

### 3.2. Materials of Electrodes

The electrode material is essential for the sensitivity and selectivity of an impedance measurement system. The selection of the material mainly depends on the purpose of use, the inertia of the material to the environment, the complexity of the manufacturing process and the cost. In order to simplify the manufacturing process, in general, only one material is adopted for an electrode. In the application of electrophysiological signal measurement, the electrode materials commonly used are Ag/AgCl, Au, metallic nanomaterials, and carbon-based nanomaterials [44].

#### 3.2.1. Ag/AgCl

Ag/AgCl electrodes are widely used in the detection of bioelectrical signals, due to their low cost, simple and mature fabrication process, stable chemical properties and low compensation voltage. If there is no special requirement in size, the Ag/AgCl electrode will be given priority in electrophysiological signal measurement. The form of the Ag/AgCl electrode is different according to the application purpose and can be a dry electrode, disposable pre-gelled wet electrode or an electrolyte electrode [51].

#### 3.2.2. Au

The gold electrode has the advantage of stable chemical properties, excellent biocompatibility and good conductivity. However, as one of the precious metals, considering the cost factors, gold is commonly used in micro electrodes [52]. The use of a gold electrode for skin impedance is suggested to be as an electroplate on a flexible substrate like polyimide and thin silicon to allow good attachment to the skin.

#### 3.2.3. Nanomaterials

Nanomaterials are frequently adopted in sensors for physiological measurement due to advantages like a large surface area and good connectivity and electrical conductivity [53,54,55]. The commonly used nanomaterials include metallic nanomaterials and carbon-based nanomaterials [42,56].

Metal nanoparticles and metal nanowires are very popular in the application of dry electrodes. AgNW embedded in polydimethylsiloxane (PDMS) was used as an example to introduce the properties of metal nanomaterials [42]. These electrodes are highly stretchable and have a conductivity of ~5000 S/cm at 50% tensile strain. When the subject is in a resting state, the performance of the AgNW/PDMS electrode is similar to that of the Ag/AgCl electrode, while the performance of the former is better in moving states. In addition, the material was tested in a biocompatible experiment.

Carbon nanotubes (CNTs) have good mechanical strength, good conductivity, are low cost, and are convenient for mass-manufacture. Carbon nanotubes can be added to the polymer matrix to make the material softer and more conductive. A high concentration of carbon nanotubes has a large contact area, which can improve the quality of signal acquisition. Previous experiments have shown that its conductivity is still good at 45% tensile strain. It was found that the existence of sweat and long-term wearing has little influence on the performance of the electrode through the comparison between its use in the steady state and moving state. In addition, the skin did not present any allergic reactions to the electrode after a 7-day toxicity test [56].

However, there exit some potential security risks for the use of nanomaterials. Toxic effects of nanoparticles (NPs) perniciously affect the normal structures of tissues or organs due to the size and shape of NPs [56,57]. According to the results of existing studies, there is no direct evidence to prove NPs are either harmful or harmless [57]. However, the nanomaterial-based electrodes for SC impedance detection are non-invasively attached to the surface of the skin, which is supposed to have little influence on normal physiology.

### 3.3. Size and Geometry of Electrodes

The size and geometry of an electrode can determine the effective contact area, signal to noise ratio, and sensitivity, especially for skin impedance information from the microscopic aspect. Reference [58] pointed out that the geometry and size of an electrode will affect the depth and intensity of the electric field. In order to get more information about the shape and size of electrodes, some relevant papers from recent years are reviewed in this section.

According to the literature, the shape of the electrodes used for skin impedance measurement can be rectangular, circular, spiral, interdigital or a concentric ring [51]. Among them, the rectangular and circular electrodes are usually macro block electrodes of relatively large size, while the other three kinds are commonly micro size electrodes. The spiral and interdigital shapes are designed to increase the effective contact area. The concentric ring electrode could exit in the form of an electrode matrix, which is convenient for shifting the electrode spacing.

Without considering the influence of electrode size, researchers generally use the rectangular or circular electrode, such as in the measurement of skin EMG [51] and in studies of the influence of chemical treatment on skin impedance [20]. However, its macro-level size means that it cannot detect specific skin layers because of its deeply distributed electric field. Thus, when used for obtaining micro-level information of organisms, the electrode should be changed to micro-size which is similar to the size of skin thickness [27,40]. Gold spiral electrodes and interdigitated electrodes are the commonly used micro-electrodes. Reference [41,52] designed a gold spiral electrode and found its size affected the impedance results, and it can also be affected by a subject’s characteristics. Reference [59] conveyed research about an interdigitated electrode. It was shown that the interdigitated electrode can be used for the detection of physiological signals and the study of different materials, due to its high sensitivity, simple fabrication process and mature theoretical analysis model.

It was verified in [44] that changes in electrode spacing have little effect on skin impedance when the electrode spacing is greater than one centimeter. In reference [40], researchers pointed out that the electric field distribution of electrode is related to the size of the electrode itself and the electrode spacing. A few researchers took the interdigital electrode as an example and analyzed the influence of electrode size on the distribution of electric field and the penetration depth [60]. It was shown that the electrode spacing and the two fingers width ratio have the most significant impacts on the signal sensitivity, while the height affects the signal-to-noise ratio greatly. The function of interdigital electrodes, as pointed out in reference [59], is mainly based on the fringing electric field between the two fingers. The finite element analysis method was used in their study to determine how the electrode spacing affects the electric field distribution. The results showed that with an increase in electrode spacing, the electric field was more deeply distributed and the electric energy density became smaller.

The electric field of an interdigital electrode was simulated by the program QuickField [40]. It was found that the electric field is mainly concentrated in the distance between the fingers. Therefore, the electrode spacing size must be designed and fitted to the size of the thickness of the target skin layer when the skin impedance of a specific layer is measured. Reference [59] drew the conclusion that the interdigital electrode is suitable for detecting the properties of multi-layers of different materials without damage. Furthermore, the skin structure is similar to the stacking of three layers of different materials. Thus, the interdigital electrode can be very useful in the impedance measurement of different skin layers. Besides the electrode spacing factor, there have also been studies regarding the effect of the finger width ratio on the electric field distribution. Electrodes whose two fingers’ width is not equal are called non-symmetric electrodes. It was shown that if the two fingers’ width ratio is more than or equal to 3, the field strength is 30% higher than that of a symmetric electrode, and 80% of the electric field falls on the finger-spacing size of the thickness of the electrode spacing. It could be deduced from these results that the size and geometry of the electrode have a crucial impact on the experimental results when the size of the electrode is at the micro-level.

Above all, different electrodes have different properties which were listed in Table 3. The size of an electrode has greater impact on the impedance measurement than other factors, according to previous literature. Thus, there exists potential opportunity for multi-sensing between the SC impedance measurement and other wearable electrochemical sensors. Furthermore, an electrode could be reused for the two measurement processes by adjusting the measurement system. However, further research is required to determine whether different processes will adversely affect the overall performance.

## 4. Methods and System for Impedance Measurement

A skin impedance measurement system is determined by three parts: detection site, electrode and measurement system. The detection site commonly chosen is the side abdomen of the human forearm or rat abdomen because these sites are thin and easy to access [61]. The number and layout of electrodes are different according to the requirements and principles. The measurement system needs to provide a stable excitation signal, be sensitive to response signals and achieve a certain amplification and filtering function so as to get an ideal final signal.

### 4.1. Methods for Impedance Measurement

Skin impedance, like other biological impedances, varies with frequency. Therefore, the measurement method of skin impedance is mainly based on electrochemical impedance spectroscopy (EIS). At present, the EIS method has been widely used in some clinical studies, such as in transdermal drug delivery by measuring skin impedance changes to determine the absorption of drug delivery, in monitoring of the late recovery of osseointegration in hearing therapy, and in studying muscle state [11]. Besides the EIS method, another method which means skin impedance measured at a single low frequency, such as f = 100 Hz or f = 1000 Hz, has also been adopted in research of the relationship between skin impedance and skin permeability [17,22].

Normally, there are four main measurement layouts of electrodes to obtain skin impedance including the four-electrode array, the two-electrode method, the three-electrode method and the tip-electrode method [11].

**Figure 6:**
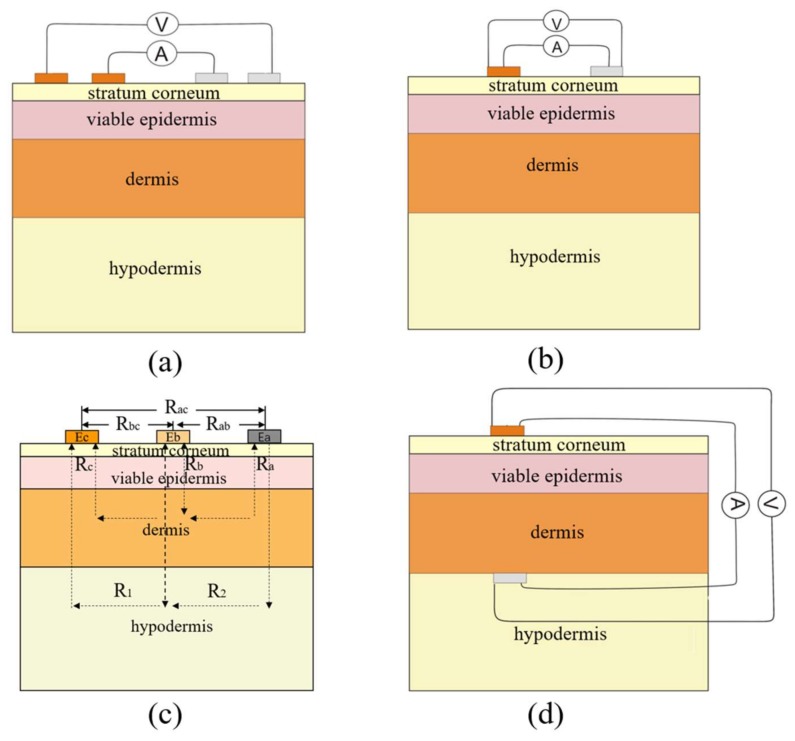
Four different measurement layouts of electrodes: (**a**) shows the layout of the four-electrode method, the inner two electrodes are response signal detection electrodes and the outmost two electrodes are stimulus signal electrodes; (**b**) represents the layout of the two-electrode method, the detection electrodes and the stimulus electrodes are multiplexing; (**c**) represents the layout of the three-electrode method, the skin is regarded as pure resistance [62]; (**d**) represents the layout of the tip-electrode method, the most direct and accurate way to detect skin impedance, but it is invasive.

The four-electrode array is commonly used in biological impedance measurement systems and has two excitation electrodes outside and two detection electrodes inside (Figure 6a). This method is appropriate for impedance detection over a wide frequency range and could reduce the influence of the contact resistance of the electrode. Besides, this method is only used to measure the skin impedance between two points. If the detection area is small enough, such as in a small penetration treatment site, the excitation electrodes and the detection electrodes could be reused to realize the two-electrode method (Figure 6b). Therefore, the two-electrode method is actually a simplified four-electrode array method. In two-electrode systems, non-symmetric interdigital electrodes are supposed to perform better than symmetric ones because the electric field of non-symmetric interdigital electrodes concentrates more in the skin surface layer. 

The skin impedance model for the three-electrode method is shown as Figure 6c; it is a simplified model and only considers the resistance property of skin. E_A_, E_B_ and E_C_ represent the three electrodes—A, B and C—respectively. Electrode B is located on the right, above the target skin area. R_A_, R_B_ and R_C_ are the resistances of the epidermis in three corresponding positions. R_1_ is the resistance of tissues below the epidermis between B and C, while R_2_ is that between A and B, where the tissues below the epidermis refer to the whole of the dermis and the subcutaneous tissue. R_AB_, R_BC_ and R_AC_ represent the detectable resistances by two electrodes out of the three. The resistance of target position B could be deduced by Formula (3), when R_AB_, R_BC_ and R_AC_ are measured.

(3) $$\begin{array}{l} R_{B}=((R_{A}+R_{2}+R_{B})+(R_{B}+R_{1}+R_{C})-(R_{A}+R_{2}+R_{1}+R_{C}))/2\\ \;=(R_{\mathrm{AB}}+R_{\mathrm{BC}}-R_{\mathrm{AC}})/2 \end{array}$$

The layout of the tip-electrode method is shown as Figure 6d, which has a tip-electrode placed below the target position of the skin and the other electrode placed above the same position on the skin [18]. This is the most direct method for skin impedance measurement but is inconsistent with the original intention of non-invasive disease monitoring through transdermal ISF extraction or transdermal drug delivery.

The above-mentioned four kinds of impedance measurement layouts all can be used to measure skin impedance. However, only the two-electrode method can achieve the requirement of SC impedance measurement to non-invasively access the skin permeability intention in this paper, to gain enough information to distinguish skin differences between individuals. In addition, the two-electrode method is more suitable for integrated portable wearable devices. Traditional skin impedance measurement methods were based on macro-electrode, which is not ideal for measuring the impedance of SC. The electric field could only be generated into the SC layer when the distance between macro-electrodes was down to the size of the skin layer [40]. Considering the frequency characteristics of different skin layers and the properties of micro-electrodes, an improved two-electrode method could perform better for SC impedance measurement. Firstly, a micro-electrode should be designed with the appropriate size and structure to concentrate the working electric field into the target layer. Secondly, the measuring frequency should be controlled below 1000 Hz, which is suitable for SC frequency characteristics.

### 4.2. System for Impedance Measurement

In the 1990s, the early impedance measurement system was introduced as the following parts: it contains a Macintosh, in which the signal generator output a sinusoidal excitation signal with a frequency ranging from 1 Hz to several kHz, controlled by LabVIEW3.0.1. The electrical circuit used for the system included a 2-MΩ resistor in series with the skin. Thus, the sinusoidal current remained approximately constant at 0.2 µA. Eight or ten frequency points were sampled per period. The response voltage of detected skin was measured by a lock-in amplifier. An isolation transformer was used to ensure complete electrical separation of the subject from the main power supply, so as to protect the human body [44].

Over the past 10 years, microcontroller unit (MCU) and digital signal processing (DSP) technology has made great progress and the demand for better accuracy of the bio impedance technology has driven the impedance measurement system in a more integrated and systematic direction. The upgraded measurement system contains three main parts: (1) a notebook computer, providing software for the user interface and data processing; (2) a multifunctional I/O conversion board (such as: PCMCI, DAOCard6062E), generating an excitation signal and collecting response signals under the condition of electrical insulation; (3) an analog interface module, including a differential amplifier to ensure high noise immunity and common mode rejection ratio (CMRR), which is necessary for biological electrical detection at low frequencies [63].

There are some commercially available devices for performing EIS methods, like Gamry (Warminster, PA, USA) and Autolab (Herisau, Switzerland) [17,64], which perform well in accuracy and efficiency. However, these instruments are too large to be used for portable application. With the development of integrated chip technology, the authors of study [65] used a single chip AD5933 to measure complex impedance. This single chip could generate the excitation voltage signal and detect the response current signal under the condition of complete electrical separation, in addition to processing signal filtering and amplification. The measuring range of AD5933 is 1 kΩ~10 MΩ; the range could be extended to 100 Ω~10 MΩ with an appended circuit. In additino, a high measurement accuracy could be realized with an output frequency between 1 kHz and 100 kHz. Its sampling rate is 250 kSPS [65]. Above all, this chip achieves a higher resolution and a wider range of measurement frequency, so that we can get more accurate impedance information to capture small impedance changes. Therefore, AD5933 allows the miniaturization of the measurement system.

Lots of work has been done in regard to wearable sensors in the domains of environment monitoring, fitness, and healthcare [48]. Wearable devices for fitness and healthcare could be combined with SC impedance measurement to optimize their performance and improve their practical application value among different individuals. Wearable devices are becoming more lightweight, thin, multi-functional, and with a more reliable performance. Researchers have devoted much effort to the development of a flexible sensing platform for healthcare monitoring, electronic skin and soft robotics [66,67,68]. Rapid advances in flexible sensors and sensing platforms in recent years have shown the growing importance and feasibility of this technique. The impedance detection system, as a feedback module for certain types of wearable equipment, should preferably be in the form of flexible type, which is more comfortable for subjects to wear for a long time. However, in order to make the envisioned flexible sensing system closer to real application, much more attention should be drawn to factors like seamless integration to data processing and wireless communication modules, in addition to functional sensing modules.

In the future, skin impedance measuring instruments are predicted to develop in the direction of miniaturization, multifunction, speediness and intelligence. They is expected to be based on the flexible sensing platform and wireless transmission technology so that the skin impedance measurement is flexible enough to be integrated into other instruments as an auxiliary function. Besides, the smartphone is a useful and portable terminal device to display measurement results for wearable sensors in the healthcare domain [69]. Therefore, based on Bluetooth transmission technology, the smartphone could be used to display SC impedance detection results instead of any other external electronic dongle. This would make a great contribution to the fields of mobile healthcare and wearable devices.

## 5. Application in Penetration Promotion Treatment

Skin impedance can characterize the status of ion channels inside the skin to some extent. If the number of the ion channels is enough, or the path is big enough, ions can be more easily transported to the skin surface; the skin impedance will, in turn, be smaller, which means the percutaneous ISF extraction will be easier. Thus, the skin impedance, especially the impedance of SC, could not only be used in the distinction of individual differences, but also as a reference for assessing the effect of penetration promoting. In this module, two penetration treatment methods will be introduced to show the potential for assessing the effect of penetration treatment with SC impedance measurement.

### 5.1. Iontophoresis

**Figure 7:**
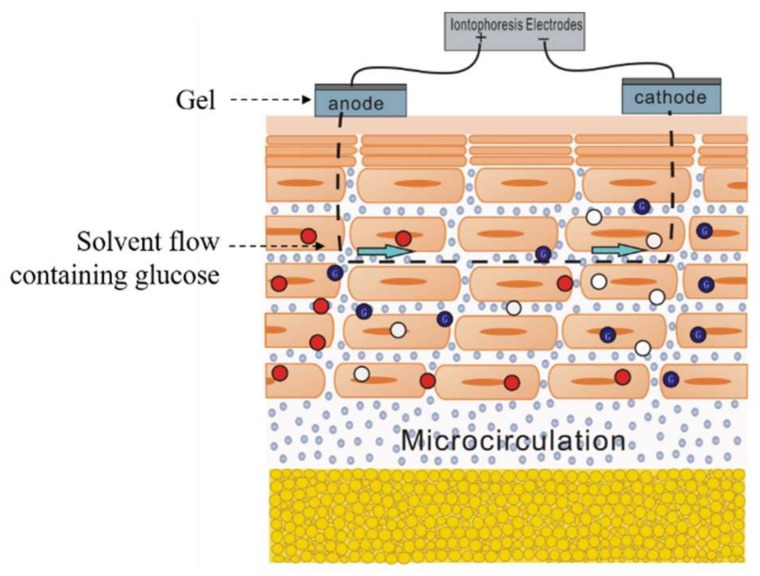
Schematic illustration of the principle of reverse iontophoresis showing an iontophoresis extraction device supplying a constant current to an anode and cathode.

Iontophoresis is a kind of impendence measurement application. It needs two conductive electrodes, placed on the target position of the skin, and a small voltage applied to produce a low current. The final excitation current signal should generally be controlled in the 300~500 µA/cm^2^ range. The schematic diagram of iontophoresis is shown as Figure 7. The skin is a permselective membrane that, at physiological pH (~5.0–6.0), supports a net negative charge [12]. This momentum causes a net convective flow from anode to cathode. Therefore, the charged ions and uncharged molecules mainly move in that direction. In the application of transdermal ISF extraction, some researchers changed the polarity of the two electrodes periodically so that the target molecules could be transported to the skin surface continuously.

Ions are driven by the electric field of iontophoresis and move to the skin surface. The net convective flow brings ISF to the skin surface continuously, which promotes the hydration of SC. Some tissue fluid fills the intercellular path among the hydrophobic SC, which makes it easier for the ions to move through SC; the impedance will, in turn, decrease. In reference [14], the changes in the internal micro-structure of the skin before and after the treatment of iontophoresis were compared under a microscope. It was found that there were two main obvious changes. On one hand, the granular, links between different cells became loose which caused the corneocyte detachment. On the other hand, many water pools occurred in the skin beneath the electrode, especially under the anode electrode. The former helps to weaken the compact structure of SC while the latter helps to promote the hydration of SC. Therefore, the measurement of SC impedance could be used to judge the effects of iontophoresis treatment.

### 5.2. Electroporation

Electroporation technology, which originated in the United States in 1991 [70], is another application of penetration treatment. It was reported for the first time in 1993 that the percutaneous penetration amount of calcein increased by four orders of magnitude following electroporation treatment [71]. Since then, the study of electroporation in transdermal drug delivery has been deeply researched. Different drugs have been tested to check the effect of electroporation in the transcutaneous permeation field.

**Figure 8:**
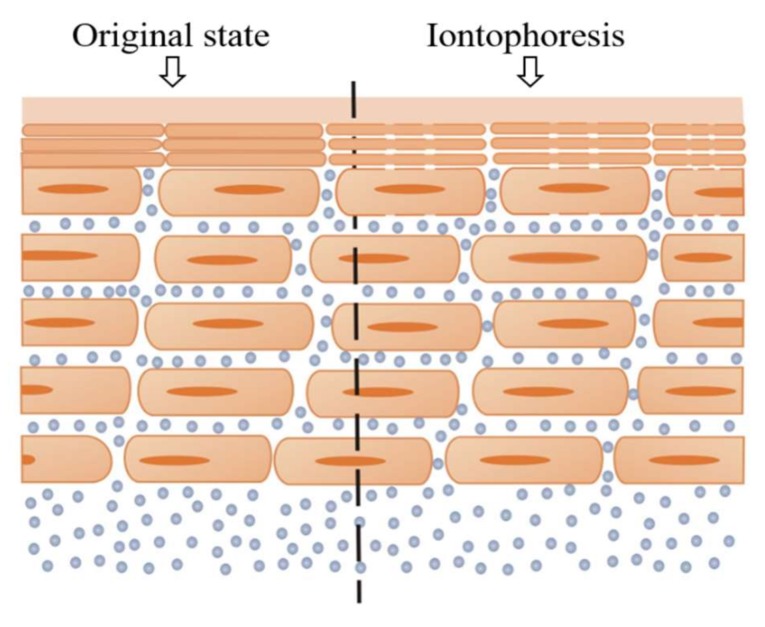
Schematic illustration of electroporation showing that new channels were made after the electroporation treatment.

Electroporation refers to the treatment of the target skin surface with electrical stimulation through a high-intensity, pulsed voltage wave (Figure 8). The pulse width and intensity both impact the stimulation effect. In reference [72], researchers proposed that new water channels would appear through the lipid bilayer in SC after electroporation treatment, and these channels would have three characteristics: (1) instantaneous, generated in the pulse period; (2) reversible, the channels would disappear after a period of time and the natural barrier function of the skin could be restored by itself; (3) hydrophilic, hydrophilic substances could pass through these channels easily.

The optical microscope and the electron microscope cannot be used to observe dynamic changes in the skin structure, so that the concept of the water channel is unable to be directly proven, and its existence can only be proved by indirect evidence. Indirect evidence is provided by the change in skin impedance—a greater stimulation intensity or longer handling time will lead to greater destruction of SC and smaller impedance, due to the increase of the density of the water channels. Therefore, SC impedance can also be an effective way to characterize the effect of electroporation.

According to the relationship between the skin impedance and the two penetration treatment methods mentioned above, we can deduce that the penetration effect and permeation ability could be reflected by SC impedance. A few researchers have indicated that skin properties (such as the thickness and hydration) of different individuals are not the same [25], which means it is difficult to extract the same amount of ISF from different individuals. This is one of the main reasons why non-invasive blood glucose detection is not accurate enough. Therefore, if SC impedance is determined as a reference criterion, we would take different penetration methods and vary their intensity for individuals with different SC impedances, until the impedance detected from all individuals reached an ideal standard. Following the extraction of ISF after such pretreatment, the accuracy of quantitative ISF extraction would be greatly improved.

## 6. Conclusions and Prospects

The development of skin impedance measurement technology was reviewed in terms of four aspects in this paper. Firstly, various skin impedance models were analyzed. Secondly, the characteristics of exiting skin impedance detection electrodes, including types, materials, size and geometry, were summarized as well as the limitations and advantages of each electrode. Thirdly, the development of measurement methods, based on different principles was also reviewed. Finally, the relationship of skin impedance with two commonly used penetration promoting methods was analyzed from the aspect of skin physiological changes. Based on the literature review, several significant conclusions and future directions are summarized, as follows:

- According to the biological structure and frequency response characteristics of the stratum corneum, SC is considered to be the key factor which affects skin transdermal transport ability. Thus, the impedance detection of SC is predicted to be an effective method for assessing skin permeability.
- The future skin impedance model is proposed to be able to express both the layer structure and biological characteristics.
- Micro-electrodes of appropriate size and structure can help to concentrate the working electric field into the target skin layer, which will improve the accuracy of impedance detection.
- Non-symmetric interdigital micro-electrodes are proposed to perform better than symmetric ones because the electric field strengths of non-symmetric electrodes are higher than that of symmetric ones.
- Impedance detection sensors and systems will develop to be more integrated, portable and flexible (skin-like), so as to be more appropriate for long-term and continuous healthcare use.
- Skin impedance could be used as feedback reference for adjusting the stimulation intensity and time for the penetration promoting method to reduce the differences between individual skin permeability.
- The technology of the skin impedance measurement and analysis method could be applied in the fields of cosmetology, dermatological research and so on.

It is expected that an improved skin impedance measurement method could be obtained for accessing the skin permeability of different individuals, which would greatly promote the progress of non-invasive chronic disease monitoring technology (like diabetes, gouts and so on) and transdermal drug delivery. The challenge is that the skin conditions greatly vary in different individuals—that is, the appropriateness of the model and micro-electrode cannot be suitable for everyone. Therefore, much work should be done to promote the theory and method of skin impedance measurement.

## Figures and Tables

**Table 1:** Comparison between different skin layers.

	Stratum Corneum	Viable Epidermis	Dermis
**Composition**	Corneocytes embedded in lipid matrix, tightly stacked and overlapping each other	Stratum lucidum, stratum granulosum, stratum spinosum, and stratum basale.	Papillary layer, reticular layer, blood vessel, lymph and nerve system
**Hydrophilicity**	no	yes	yes
**Impedance**	>$10^{5}$ Ω	>$10^{4}$ Ω	>$10^{4}$ Ω
**Thickness**	Tens of microns	150 μm	500 μm~2 mm

**Table 2:** The characteristics of two impedance models.

	Constant Phase Angle Model	R-C Layered Model
**Characteristics**	Based on the Cole model and RC parallel model, Z_cpe_ is used to characterize its biological property	Based on the layered structure of skin, model is constructed in each layer
**Parameters**	3	>20
**Accuracy**	Not accurate due to the rough model	Ignoring the biological nature of the skin
**Computation**	Small amount	Very large amount

**Table 3:** Comparison of Characteristics for Different Electrodes.

Electrodes		Type	Attachment	Size	Advantages	Disadvantages
Materials	Geometry					
**Ag/AgCl**	Bare block electrode	Dry	poor	2.25 cm^2^~10 cm^2^	Non-disposable, simple and mature fabrication process	Poor attachment
	Pre-gelled electrode	Wet	good	0.79 cm^2^/6 cm^2^	Attaches well to skin	Disposable, easily changed by sweat, not suitable for long time use
	Electrolyte electrode	Wet	good	UM ^1^	Constant humidity and attaches well to skin	Disposable, skin overhydration would greatly change skin impedance
**Au**	Spiral electrode	Dry	poor	UM	Non-disposable, improves the effective measurement area and is micro-size	Poor attachment
	Concentric ring electrode	Dry	poor	UM	Non-disposable, simple fabrication process, uniform electric field and distance could be shifted easily	Poor attachment
	Interdigital electrode	Dry	poor	a = b = d = 100 µm	Non-disposable, mature theoretical analysis model, widely used	Non-uniform electric field distribution
**Nanomaterials (AgNW, Au, CNTs)**	Based on the substrate material Polydimethylsiloxane (PDMS), polymide, and textile)	Dry	poor	UM	Non-disposable, biocompatible, stretchable, performs well in the moving state, good mechanical strength, and large contact area	Complicated fabrication process and high cost, only used in laboratory.

^1^ UM: unmentioned.
